# Maxillonasal dysplasia (Binder's syndrome) and its treatment with costal cartilage graft: A follow-up study

**DOI:** 10.4103/0970-0358.44930

**Published:** 2008

**Authors:** Mukund Jagannathan

**Affiliations:** Professor and Head, Department of Plastic Surgery, LTMG Hospital, Sion, Mumbai, India

The term nasomaxillary hypoplasia was first used by Converse[1] in 1970, to describe a variety of clinical conditions, which have in common, a significant underdevelopment of the nasomaxillary complex. Henderson and Jackson[2] in 1973 further classified the deformity clinically, based on the involvement of the dentoalveolar segment.

Regarding the nasal pathology, Rintala[3] has described two types of nasal deformities: flattened nose of normal length, and a foreshortened nose. Jackson et al[4] have described the columella and upper lip as being indrawn into the nasal floor, and lack of palpability of the nasal spine and the pyriform fossa.

There are several options for treatment depending on the degree and the severity of the deformity. The various components of the deformity are addressed individually or in combination.

- Nose:
  Nasal lengthening (skin, cartilage and septum)
  Columellar lengthening
  Dorsal augmentation
  Tip projection
- Maxillary platform augmentation
- Inlay grafting
- Osteotomies

The authors have shown fairly acceptable results in their cases. However, one of the cases was very mild, and did not have typical features of nasomaxillary hypoplasia, but rather more of a depressed nasal dorsum and underprojecting tip (Figures 8 and 9in the article). The more difficult cases ideally needed osteotomies to bring the nose and perinasal area forwards. Without osteotomies, despite onlay grafting, the result will always be compromised. Tip grafting may be additionally needed.

I agree with the authors that one need not wait for skeletal maturity to operate these patients. However, one should warn the patient about the possibility of repeat surgery.

As far as exposure of the nasal framework is concerned, it is not strictly necessary to take an external (in their cases- midcolumellar) incision. It is entirely possible to deglove and skeletonise the nasal framework with a buccal sulcus incision and transfixion incision continuing as an infracartilaginous incision.

When the deformity is severe, bone grafting allows a greater correction of the sunken nose. Bone grafts must be cantilevered, or used as a L shaped structure. This provides mechanical support to maintain the tip in place. Cartilage grafts on the other hand cannot be used as stress bearing structures. They are more spacers, which can allow a mild stretching of tissue.

Another issue is the limitation of the lining of the nose. In cases where the hypoplasia is extreme, the entire nasal lining may have to be released from the maxilla and a post nasal inlay as described by Gillies[5], may be needed. The patient usually has to wear a permanent prosthesis to maintain the projection. Nasolabial flaps may occasionally be used to resurface the lining. Banks and Tanner[6] have used buccal mucosal flaps to line the defect in the nasal mucosa after release. These manoeuvres will allow the nose to stay in its new position, without too much of a contracting force.

The authors have done an extensive review of the pathology and the rationale for various modes of treatment. However, one solution rarely fits all problems, and while cartilage grafting does take care of a large cross section of cases, it is by no means the only form of treatment.
